# Functional analysis of two *SLC9A6* frameshift variants in lymphoblastoid cells from patients with Christianson syndrome

**DOI:** 10.1111/cns.14329

**Published:** 2023-06-28

**Authors:** Hailan He, Huiwen Zhang, Hui Chen, Fang He, Fei Yin, Tobias Stauber, Xiaomin Zou, Jing Peng

**Affiliations:** ^1^ Department of Pediatrics, Xiangya Hospital Central South University Changsha China; ^2^ Hunan Intellectual and Developmental Disabilities Research Center Changsha China; ^3^ Department of Pediatric Endocrinology and Genetic Metabolism, Xinhua Hospital, Shanghai Institute for Pediatric Research Shanghai Jiao Tong University School of Medicine Shanghai China; ^4^ Department of Neurology Jiangxi Provincial Children's Hospital Nanchang China; ^5^ Department of Human Medicine and Institute for Molecular Medicine MSH Medical School Hamburg Hamburg Germany

**Keywords:** Christianson syndrome, lipid, lymphoblastoid cells, mitochondria, NHE6, *SLC9A6*

## Abstract

**Background:**

Christianson syndrome (CS) is caused by mutations in *SLC9A6* and is characterized by global developmental delay, epilepsy, hyperkinesis, ataxia, microcephaly, and behavioral disorder. However, the molecular mechanism by which these *SLC9A6* mutations cause CS in humans is not entirely understood, and there is no objective method to determine the pathogenicity of single *SLC9A6* variants.

**Methods:**

Trio‐based whole exome sequencing (WES) was carried out on two individuals with suspicion of CS. qRT‐PCR, western blot analysis, filipin staining, lysosomal enzymatic assays, and electron microscopy examination, using EBV‐LCLs established from the two patients, were performed.

**Results:**

Trio‐based WES identified a hemizygous *SLC9A6* c.1560dupT, p.T521Yfs*23 variant in proband 1 and a hemizygous *SLC9A6* c.608delA, p.H203Lfs*10 variant in proband 2. Both children exhibited typical phenotypes associated with CS. Expression analysis in EBV‐LCLs derived from the two patients showed a significant decrease in mRNA levels and no detectable normal NHE6 protein. EBV‐LCLs showed a statistically significant increase in unesterified cholesterol in patient 1, but only non‐significant increase in patient 2 when stained with filipin. Activities of lysosomal enzymes (β‐hexosaminidase A, β‐hexosaminidase A + B, β‐galactosidase, galactocerebrosidase, arylsulfatase A) of EBV‐LCLs did not significantly differ between the two patients and six controls. Importantly, by electron microscopy we detected an accumulation of lamellated membrane structures, deformed mitochondria, and lipid droplets in the patients' EBV‐LCLs.

**Conclusions:**

The *SLC9A6* p.T521Yfs*23 and p.H203Lfs*10 variants in our patients result in loss of NHE6. Alterations of mitochondria and lipid metabolism may play a role in the pathogenesis of CS. Moreover, the combination of filipin staining with electron microscopy examination of patient lymphoblastoid cells can serve as a useful complementary diagnostic method for CS.

## Graphical abstract

Altered mitochondrial and lipid homeostasis may be the underlying mechanism of Christianson syndrome (CS). The combination of filipin staining with electron microscopy analysis of patient lymphoblastoid cells can serve as a useful complementary diagnostic method for CS.

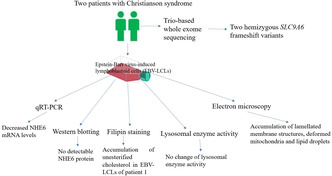

## 1 INTRODUCTION

Christianson syndrome (CS, OMIM 300243), a rare X‐linked neurodevelopmental and progressively neurodegenerative disorder, is characterized by severe global developmental delay, seizures, ataxia, microcephaly, and behavioral disorder.^1^, ^2^, ^3^, ^4^, ^5^ It was first reported in a multigenerational Caucasian South African family by Christianson et al.^1^ in 1999. Subsequently, CS was found to be caused by mutations in the human gene solute carrier family 9 isoform 6 (*SLC9A6*).^2^ To date, more than 80 distinct *SLC9A6* disease‐causing variants have been identified in CS patients, mostly with frameshift or nonsense mutations.^2^, ^3^, ^5^, ^6^, ^7^, ^8^

The *SLC9A6* gene, located at chromosome Xq26.3, encodes the endosomal Na^+^/H^+^ exchanger 6 (NHE6).^2^, ^9^, ^10^ It is expressed broadly, with highest expression in the central nervous system, especially in the hippocampus, cortex, and cerebellum,^11^, ^12^, ^13^ which likely explains the marked neurological morbidity of CS.^12^ NHE6 is predominantly localized to early and recycling endosomes.^14^, ^15^ It plays a role for the processing and trafficking of intracellular cargo, likely by regulating endosomal pH of early and recycling endosomes through exchanging luminal H^+^ for Na^+^. Moreover, NHE6 has been proposed to play roles in growth of dendritic spines and neuronal arborization,^11^, ^12^, ^13^ indicating that NHE6 is critical to normal nervous system development and function. Indeed, deletion of *SLC9A6* in mice results in endosomal‐lysosomal dysfunction as evidenced by the intraneuronal accumulation of GM2 ganglioside and unesterified cholesterol, accompanied by a progressive loss of Purkinje cells.^11^ Notably, β‐hexosaminidase activity, which is involved in the lysosomal degradation of GM2 ganglioside, was almost undetectable in GM2‐accumulating neurons.^11^ Currently, the diagnosis of CS is based on genetic testing. However, there is no method to determine the pathogenicity of single *SLC9A6* variants in clinical practice. Here, we reported two new cases of CS with previously reported *SLC9A6* frameshift variants and evaluated whether filipin staining, lysosomal enzymatic assays, and electron microscopy examination in lymphoblastoid cells may serve for the diagnosis of CS.

## 2 MATERIALS AND METHODS

### 2.1 Standard protocol approvals and patient consents

Patients were enrolled for this study after obtaining written informed consent. This study was approved by the Ethics Committee of Xiangya Hospital of Central South University, China (Human study/protocol #201603205) and carried out in accordance with the ethical standards laid down in an appropriate version of the Declaration of Helsinki.

### 2.2 Whole‐exome sequencing

Genomic DNA was extracted from peripheral blood lymphocytes from the affected individuals and their parents using the SureSelect Human All Exon V5 Kit following the manufacturer's protocol (Agilent Technologies, Santa Clara, CA, USA) and sequenced on an Illumina HiSeq X Ten (Illumina, San Diego, CA, USA) with 150‐bp paired‐end reads. Whole‐exome sequencing (WES) data analysis was performed as described previously.^16^ Sanger sequencing was used to validate variants in the two families.

### 2.3 Lymphoblastoid cell lines

Lymphoblastoid cell lines were established from peripheral blood cells by Epstein–Barr virus transformation following standard procedures. Lymphoblastoid cell lines were maintained in Roswell Park Memorial Institute 1640 medium with 10% (v/v) fetal bovine serum, 100 μg/mL streptomycin, and 100 U/mL penicillin, at 37°C in a humidified 5% CO_2_ atmosphere.

### 2.4 Real‐time reverse transcription PCR analysis

EBV‐LCLs RNA was extracted using the total RNA Kit I (Omega, R6834‐01). Real‐time quantitative reverse transcription PCR (qRT‐PCR) was performed on three independent samples from the two patients (collected at different times) and two independent healthy controls. Reverse transcription was performed using 1000 ng RNA with the Hiscript II Q RT SuperMix (Vazyme, R22301). qRT‐PCR was performed using 1/5 dilutions of cDNAs with the ChamQ Universal SYBR qPCR Master Mix kit (Vazyme, Q711‐02). Following primers were used for *SLC9A6*, forward primer 5′–CCATGGACGAGGAGATCGTG–3′ and reverse primer 5′–ATCATAGCCAGGCCGGTTT–3′, and for *GAPDH* 5′–ACAGCCTCAAGATCATCAGC–3′ and 5′–GGTCATGAGTCCTTCCACGAT–3′. Relative quantification of *SLC9A6* gene expression was evaluated in triplicate, using the *GAPDH* expression for normalization.

### 2.5 Western blot analysis

Lymphoblastoid cells were washed in phosphate‐buffered saline (PBS) and lysed in ice‐cold RIPA buffer with phenylmethanesulfonyl fluoride solution followed by sonication. Protein concentration was determined using a BCA protein assay kit (Thermo Fisher, 23,227). Cell lysates were denatured at 95°C for 10 min. Equal amounts of protein were separated by SDS‐PAGE and transferred onto polyvinylidene difluoride membrane. Membranes were blocked in 5% fat‐free milk dissolved in Tris‐buffered saline with 0.1% Tween 20 at room temperature for 1 h and subsequently incubated with following primary antibodies overnight at 4°C: mouse anti‐β‐actin (Proteintech, 66,009‐1‐ig) and rabbit anti‐NHE6 (Abcam, ab137185). After three washes, membranes were incubated with corresponding HRP‐conjugated secondary antibodies at room temperature for 1 h. The protein bands were detected by enhanced chemiluminescence (ECL; Biosharp, BL520B).

### 2.6 Lysosomal enzyme activity

Lysosomal enzyme activities were examined in EBV‐LCLs from the two patients and six unaffected healthy individuals. Homogenates were prepared by sonication of EBV‐LCLs. Aliquots of cell homogenate were transferred in a 96‐well microplate in triplicates for the enzymatic assays as described previously: the activities of β‐hexosaminidase A,^17^ β‐hexosaminidase A + B,^18^ β‐galactosidase,^19^ and galactocerebrosidase^20^ were assessed by fluorometry using substrates 4‐methylumbelliferyl‐6‐sulfo‐N‐acetyl‐β‐D‐glucosaminide (Sigma, 454,428), 4‐methylumbelliferyl‐N‐acetyl‐β‐D‐glucosaminide (Sigma, M2133), 4‐methylumbelliferyl‐β‐D‐galactopyranoside (Sigma, M6133), and 6‐hexadecanoylamino‐4‐methylumbelliferyl‐β‐D‐galactopyranoside(Moscerdam, EH05989), respectively, with excitation at 355 nm and emission at 460 nm. The enzymatic activity of arylsulfatase A^21^ was assessed using the colorimetric substrate 4‐nitrocatechol sulfate (Sigma, N7251), absorbance was measured at 515 nm.

### 2.7 Filipin staining

EBV‐LCLs from the two patients and two controls were washed with PBS and fixed with 4% paraformaldehyde for 15 min at room temperature. Cells were thereafter washed with PBS, followed by a 10 min incubation step with 1 mL glycine (1.5 mg/mL PBS) for 10 min. After washing with PBS, EBV‐LCLs were stained with 125 μg/mL filipin (Sigma‐Aldrich, SAE0088) in PBS for 2 h at room temperature. Cells were then rinsed with PBS. Filipin‐stained or unstained control cells were analyzed using a flow cytometer equipped with a 405 nm argon laser and fluorescence emission signals were measured.

### 2.8 Electron microscopy

For electron microscopy, EBV‐LCLs were washed with PBS and then fixed in 2% glutaraldehyde in 0.1 M sodium cacodylate buffer for 2 h and post‐fixed in 1% osmium tetroxide for 2 h at 4°C. Samples were dehydrated with an ascending series of ethanol, then embedded in Araldite. Ultrathin sections were cut with an ultramicrotome and stained with lead citrate. Images were acquired using a Philips CM120 electron microscope.

### 2.9 Statistical analysis

GraphPad Prism 6 software was used for statistical analysis. Data are presented as mean ± SEM. Student's *t*‐tests or nonparametric tests were used for statistical comparison and *p* < 0.05 was considered statistically significant. *P*‐values are depicted as: *p < 0.05, **p < 0.01, ***p < 0.001.

## 3 RESULTS

### 3.1 Clinical presentations

**FIGURE 1:**
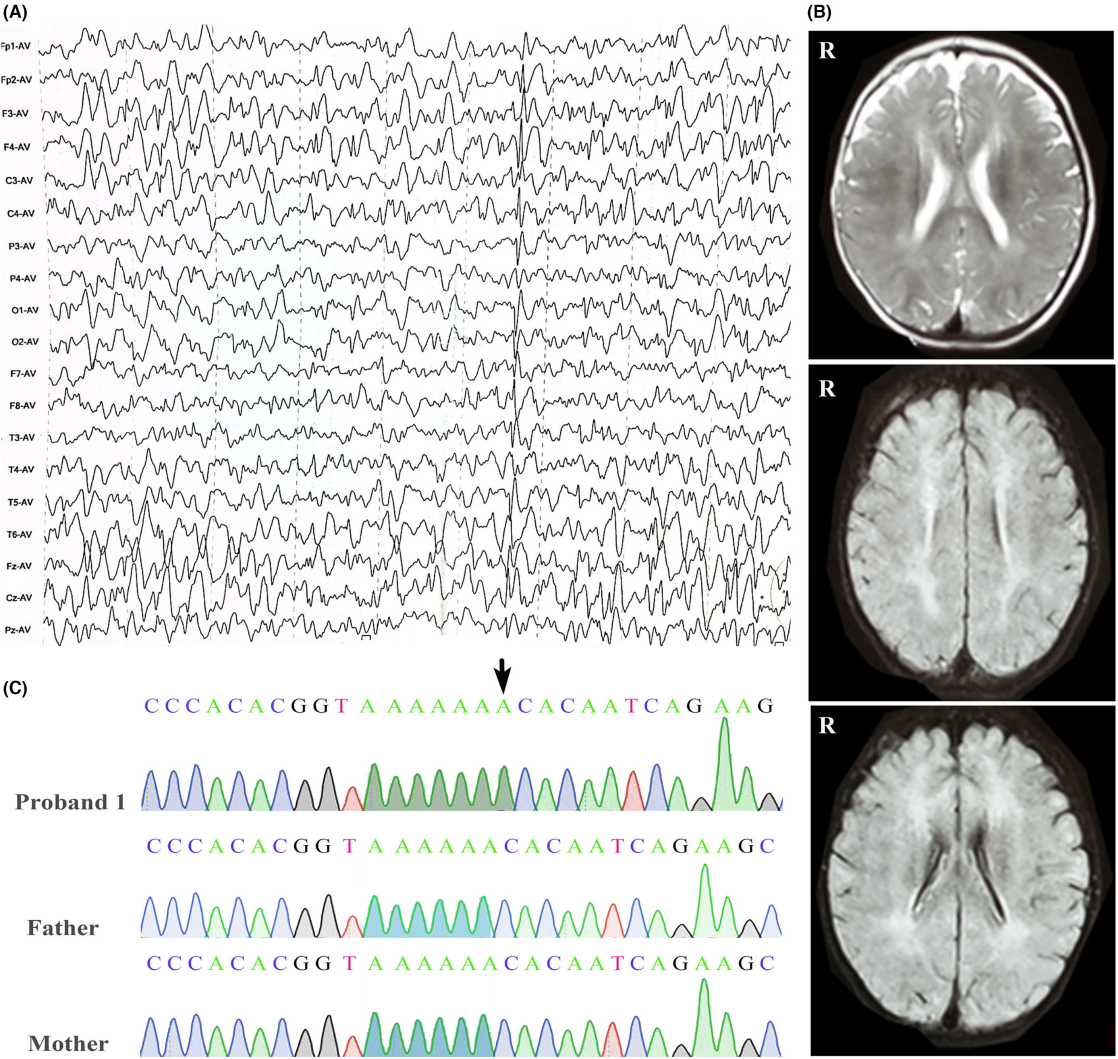
Clinical and genetic data of patient 1. (A) EEG at 1 year and 3 months showed medium or high‐amplitude sharp waves in the frontal and central region during sleep. (B) Representative brain MRI at 10 months showed FLAIR/T2 hyperintensity in the periventricular white matter. (C) Sanger sequence confirmation of the de novo single base (T) insertion in SLC9A6 (c.1560dupT, p.Thr521fs) using a reverse primer.

**Proband 1** is a 6‐year and 9‐month‐old boy of non‐consanguineous Chinese descent who was born full term by forceps delivery with suspected mild birth asphyxia. Birth weight and height were normal. There is no family history of neurological diseases. At the age of 22 months, he first showed upward gaze with consciousness loss, followed by generalized tonic–clonic seizures. Levetiracetam treatment was started without therapeutic success. He developed myoclonic seizures when he was excited at 26 months. The patient underwent vagus nerve stimulation at 5 years and 3 months, and 3 months later topiramate was added to his treatment regime but to no avail. He showed severe global development delay with head control at 1 year, sitting at 17 months of age and standing with support at 3 years old. Currently, he can walk independently but with ataxia. Language development was severely delayed with babbling at 2 years and speaking two words at 5 years. He had microcephaly, and his occipitofrontal circumference was 40 cm at 10 months (<3rd centile), and 44.5 cm at 67 months (<3rd centile). Cognitive testing with the Gesell Developmental Schedules demonstrated severe global developmental delay at 31 months of age. Electroencephalogram (EEG) showed spike wave and spike‐and‐wave discharge in multiple brain regions at the age of 10 months and medium or high‐amplitude sharp waves in the frontal and central region during sleep at 1 year and 3 months of age (Figure 1A). Brain magnetic resonance imaging (MRI) at 10 months showed FLAIR/T2 hyperintensity in the periventricular white matter (Figure 1B). Blood lactate levels were slightly elevated (2.1 mmol/L). Full blood examination, serum electrolytes, cardiac enzyme, renal function tests, liver function tests, ammonia, karyotyping, and copy number variants were normal. Trio‐based WES identified an insertion of one nucleotide (NM_001042537: c.1560dupT, p.T521Yfs*23) in exon 12 of the *SLC9A6* gene (Figure 1C), which was previously reported in a German family.^22^ No other candidate variants that may cause the disease were identified.

**FIGURE 2:**
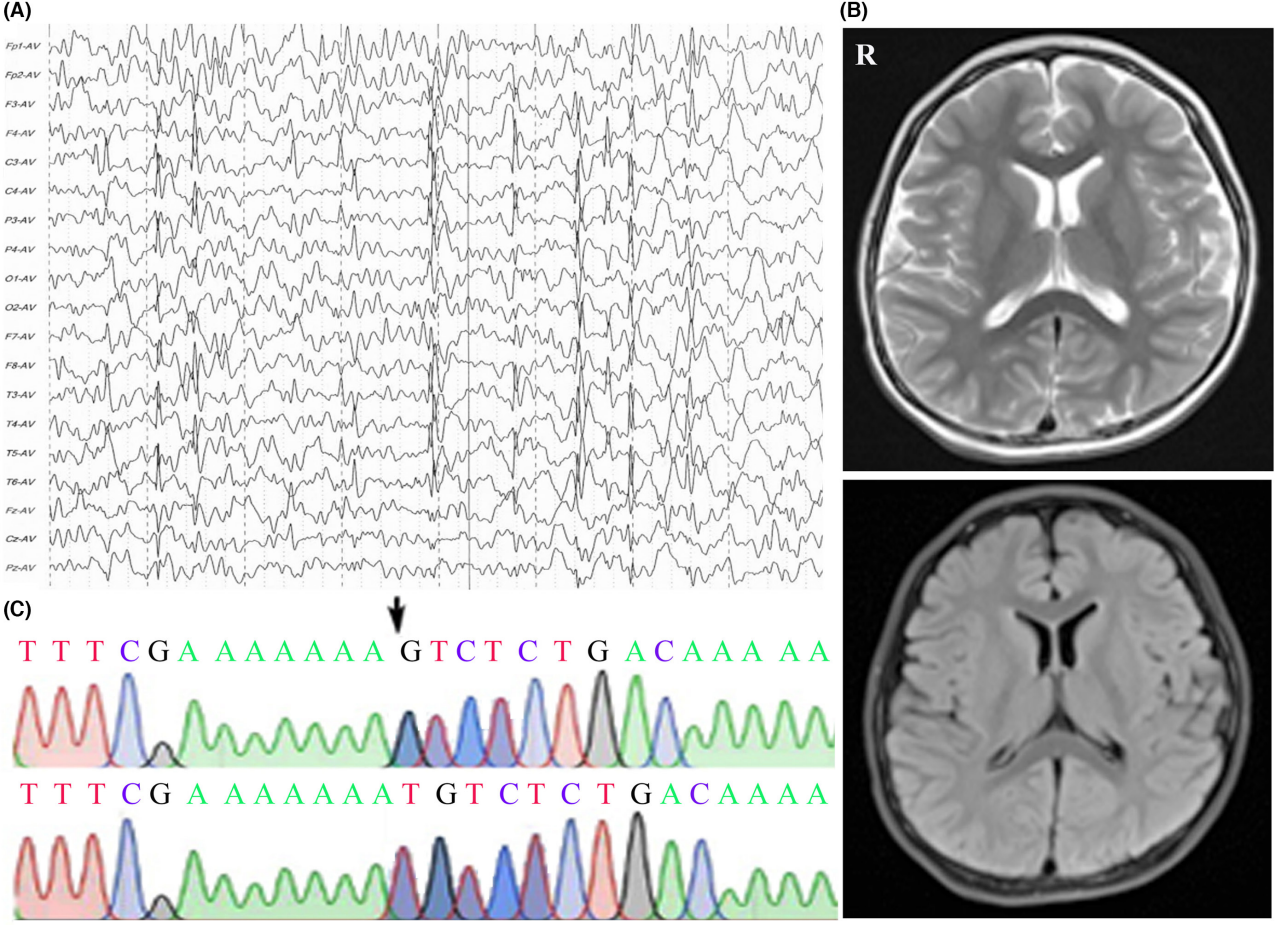
Clinical and genetic data of patient 2. (A) The EEG at 3 years and 10 months showed spike waves, spike‐and‐slow waves over the bilateral Rolandic area during sleep. (B) Brain MRI of patient 2 showed no abnormities at the age of 4 years. (C) Sanger sequencing confirmation of (c. 608delA; p. H203Lfs*10) mutation (arrow) in the index case using a reverse primer, unaffected heterozygote father (lower).

**Proband 2** is an 8‐year and 9‐month‐old boy of non‐consanguineous healthy Chinese parents. He was born at 38 weeks gestation to a 26‐year‐old mother via vaginal delivery and was 3.6 kg. He had favism and neonatal jaundice that required hospitalization for 5 days. There was no family history of neurological or neurodevelopmental disorders. He was noted to have a microcephaly with a premature closure of the anterior fontanelle at 6 months. His head circumference was 46 cm at 19 months (< 3rd centile) and 47.5 cm at 46 months (< 3rd centile). At 21 months of age, the patient started to develop intractable seizures of variable types: tonic–clonic, clonic, and myoclonic seizures, which were unresponsive to valproic acid and levetiracetam. He developed social smile at 2 months, acquired head control at 3 months, rolled over at 6 months, sat and crawled at 8 months, and started to walk unsteadily at the age of 2 years. At present, he has no speech and only can pronounce “baba” unconsciously. Gesell Developmental Schedules demonstrated moderate‐to‐severe global developmental delay at 18 months and severe‐profound global developmental delay at 59 months. He showed behavioral abnormalities with outburst of temper tantrums, flapping his hands, biting his fingers, and hyperactivity. At 22 months, multifocal sharp waves, spike waves, and spike‐and‐slow waves over bilateral anterior head areas were evident by EEG. The EEG at 3 years and 10 months showed spike waves and spike‐and‐slow waves over the bilateral Rolandic area during sleep (Figure 2A). Brain MRI revealed a narrow cranial cavity of bilateral frontal area and slight enlargement of bilateral frontotemporal subarachnoid space at 6 months and re‐examination by MRI at 4 years showed no obvious abnormalities (Figure 2B). No alternative cause for his diagnosis has been found on additional investigations including full blood examination, electrolytes, liver function tests, renal function, glucose, plasma lactate, thyroid function test, ammonia, plasma amino acids and acylcarnitine profile, urine amino and organic acids, karyotyping, copy number variants, brainstem auditory, and visual evoked potentials. By trio‐based WES, he was found to have a hemizygous deletion mutation (NM_001177651: c.608delA, p.H203Lfs*10) in *SLC9A6* that was inherited from his unaffected, heterozygous mother (Figure 2C, data for mother not shown). No other potential pathogenic findings were identified.

### 3.2 Decreased NHE6 mRNA and protein levels in patient EBV‐LCLs

**FIGURE 3:**
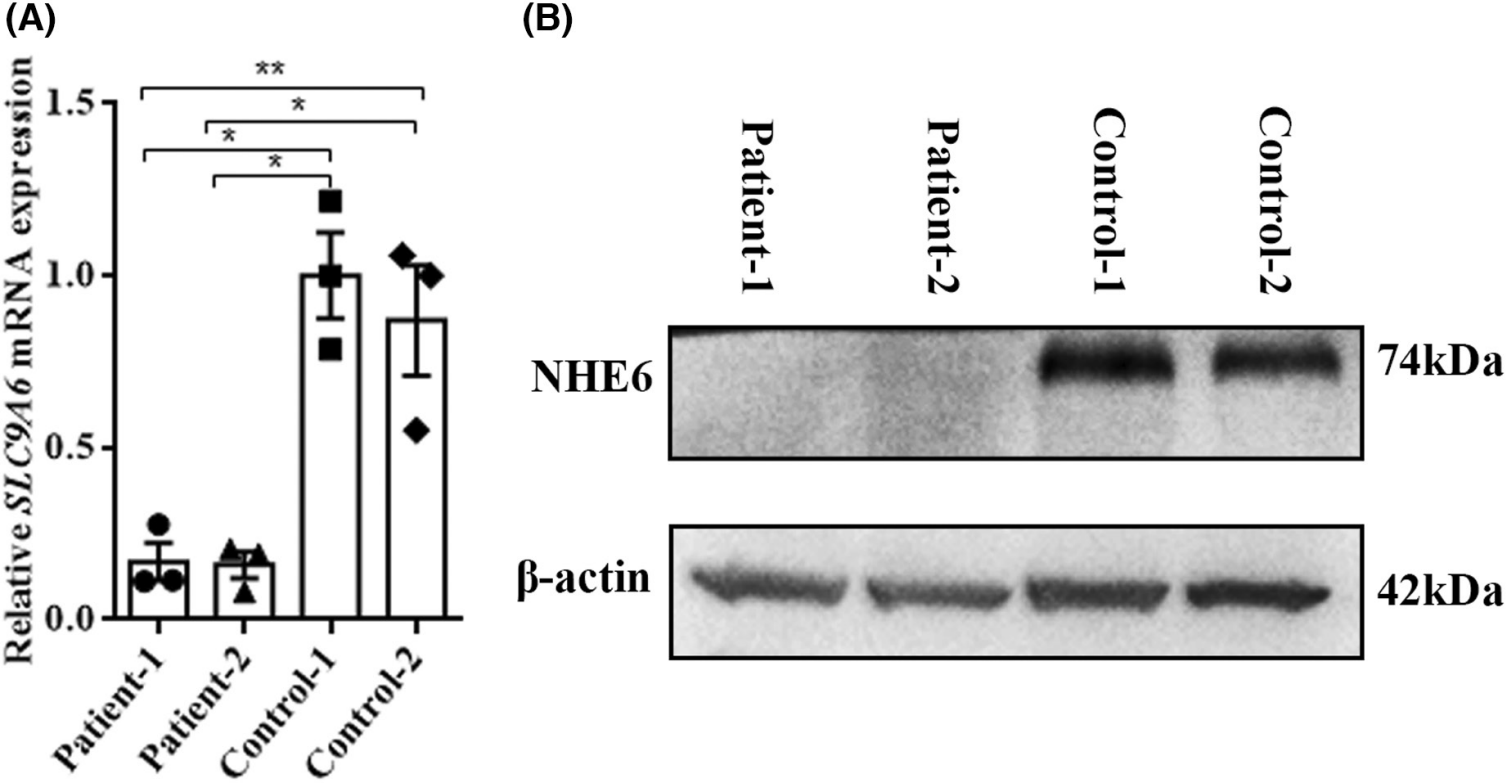
Total *SLC9A6* mRNA and protein levels of EBV‐LCLs from patients and controls. (A) Relative *SLC9A6* mRNA expression in EBV‐LCLs from patients and controls. mRNA levels were measured by qRT‐PCR and expressed as a ratio to *GAPDH*. Values are presented as mean ± SEM. Data were analyzed by Student's *t*‐tests. *p < 0.05, **p < 0.01. (B) Western blot analysis of patient EBV‐LCLs with undetectable normal NHE6 protein. β‐Actin was used as a control.

Frameshift mutations often lead to nonsense‐mediated decay (NMD) of mutant mRNA, so we first performed qRT‐PCR of total *SLC9A6* mRNA levels to assess the stability of mutant mRNA in EBV‐LCLs derived from the two patients, along with two control samples. qRT‐PCR revealed a significant reduction in mRNA for *SLC9A6* in both patients to <20% of control samples (Figure 3A), suggesting severely reduced expression of the frameshift mutated allele, probably through NMD. To assess the effect on total protein levels, homogenate of EBV‐LCLs from the patients and two normal controls were probed for NHE6 protein by western blot analysis. As expected, normal NHE6 protein was hardly detectable (Figure 3B).

### 3.3 Accumulation of unesterified cholesterol in EBV‐LCLs of patient 1

**FIGURE 4:**
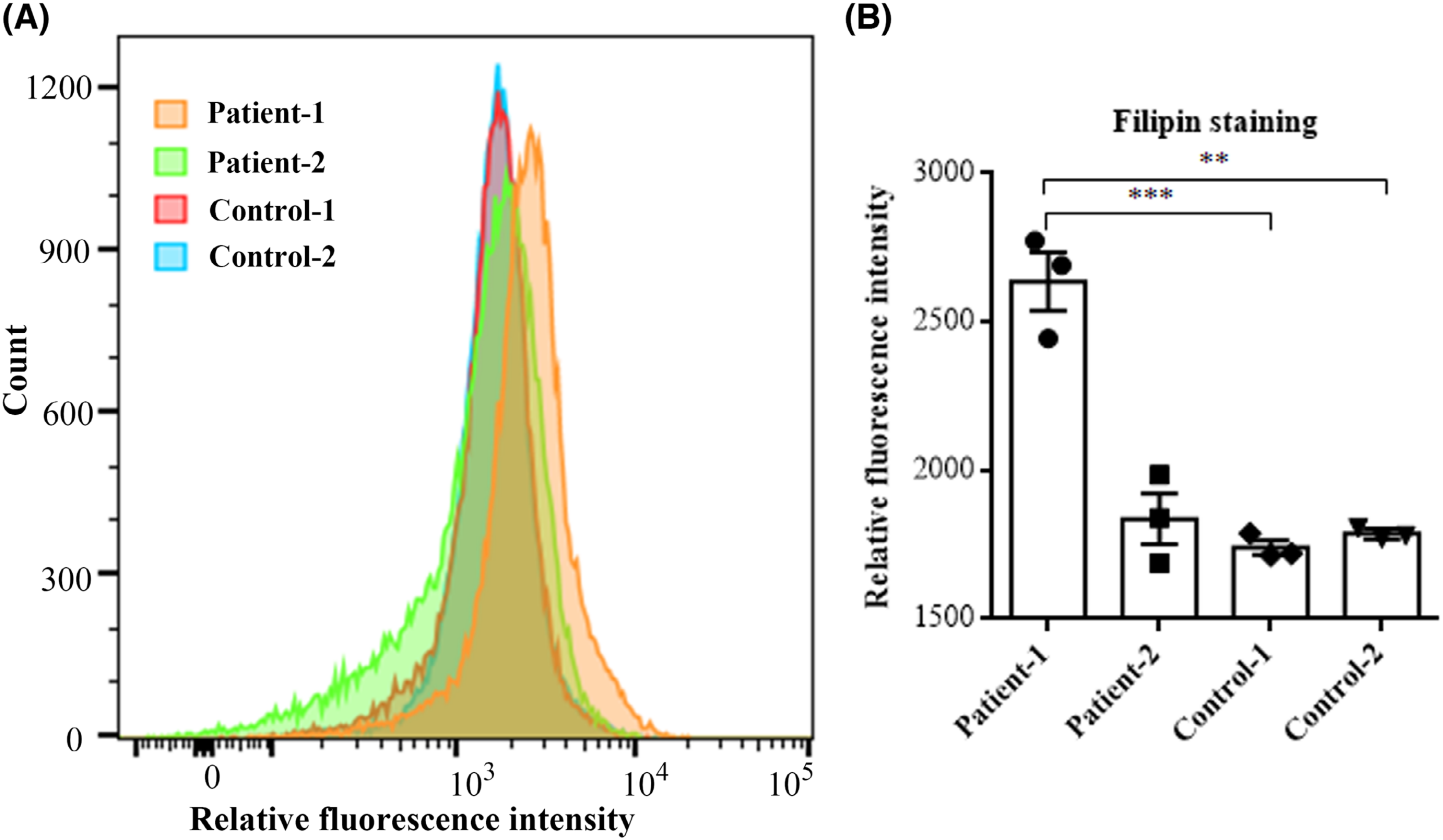
Flow cytometric analysis of fluorescence signals from filipin‐stained EBV‐LCLs from our two CS patients and two controls. Filipin staining showed a statistically significant increase in fluorescence intensity in patient 1, but only a marginal, non‐significant increase in patient 2 when compared to two normal controls. Values are presented as mean ± SEM. Data were analyzed by Student's *t*‐tests. **p < 0.01, ***p < 0.001.

The fluorescent probe filipin specifically binds to unesterified cholesterol. Unesterified cholesterol was previously shown by filipin staining in cells of the basolateral amygdala of *Slc9a6* ^*−/−*^ mice.^11^ Therefore, we assessed the levels of unesterified cholesterol in EBV‐LCLs derived from the two patients and two controls by flow cytometry after filipin staining. We observed a statistically significant increase in filipin fluorescence intensity in patient 1, but not in patient 2 when compared to two normal controls (Figure 4), demonstrating an accumulation of unesterified cholesterol in EBV‐LCLs from patient 1.

### 3.4 No change of lysosomal enzyme activity in patient EBV‐LCLs

**FIGURE 5:**
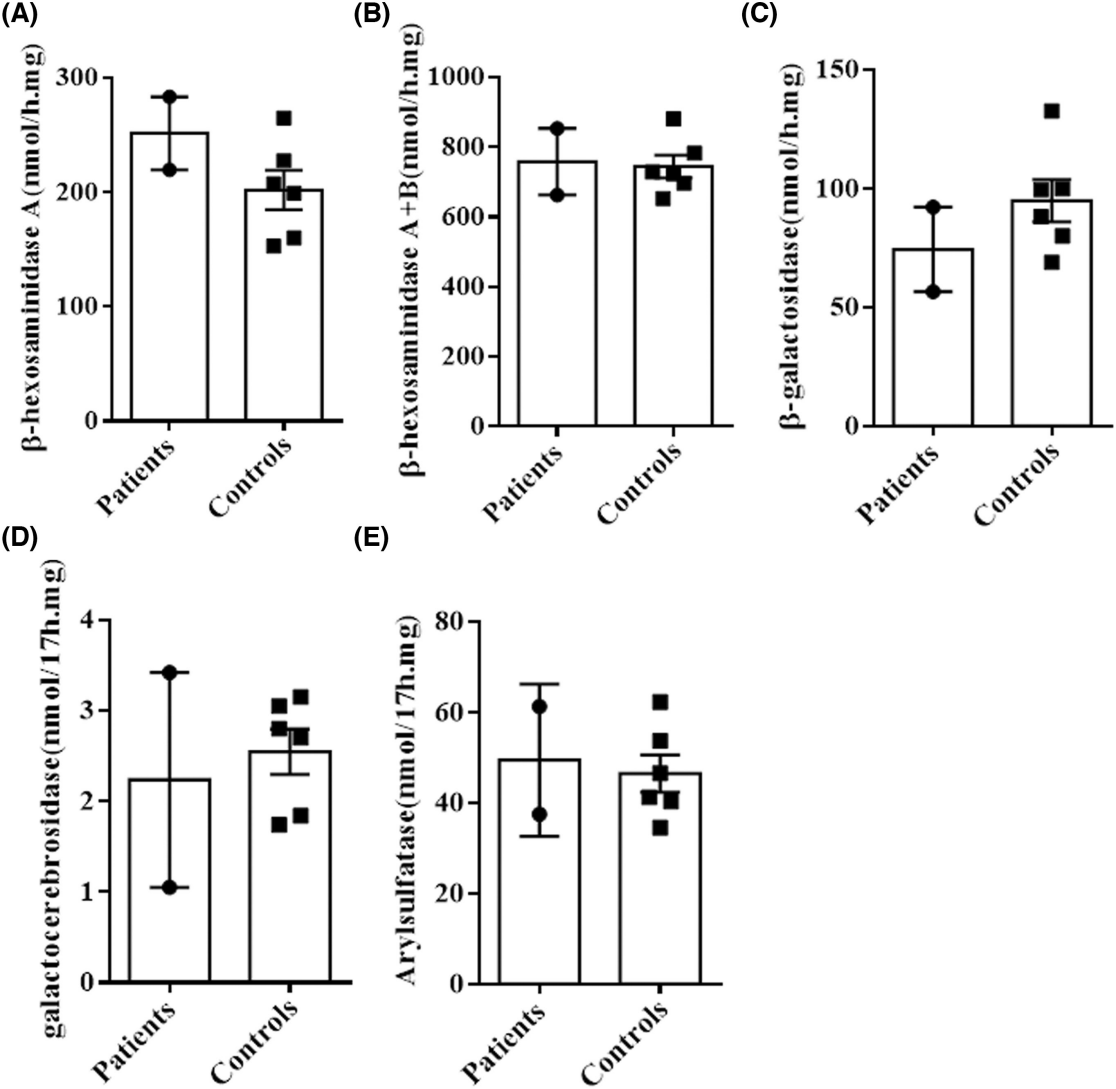
Activities of five lysosomal enzymes in EBV‐LCLs from our two CS patients and six controls. The activities of β‐hexosaminidase A (A), β‐hexosaminidase A + B (B), β‐galactosidase (C), galactocerebrosidase (D), and arylsulfatase A (E) (*p* = 0.2035, 0.8598, 0.3070, 0.6802 and 0.7613, respectively) were not significantly altered in EBV‐LCLs from our two patients. Values are presented as mean ± SEM. Data were analyzed by nonparametric tests.

NHE6‐deficient mice displayed intraneuronal accumulation of GM2 ganglioside in particular brain regions due to reduced β‐hexosaminidase activity.^11^ In order to assess the potential value of lysosomal enzyme activities in lymphoblastoid cells as diagnostic markers for CS, we examined the biochemical activities of five lysosomal enzymes in EBV‐LCLs derived from our two patients and six unaffected healthy individuals. The activity of β‐hexosaminidase A (*p* = 0.2035) and β‐hexosaminidase A + B (*p* = 0.8598), which are involved in the degradation of GM2 ganglioside, showed no obvious change in patient EBV‐LCLs compared to healthy controls (Figure 5 and Table 1). Moreover, we found no significant alterations in β‐galactosidase (*p* = 0.3070), galactocerebrosidase (*p* = 0.6802), and arylsulfatase A activity (*p* = 0.7613) in patient EBV‐LCLs (Figure 5 and Table 1). We additionally examined blood leukocytes and plasma of patient 2 for the five lysosomal enzymes but found no significant alterations (data not shown).

**TABLE 1:** Lysosomal enzyme activities in EBV‐LCLs from the two patients and six controls.

	β‐hexosaminidase A (nmol/h.mg)	β‐hexosaminidase A + B	β‐galactosidase (nmol/h.mg)	Galactocerebrosidase (nmol/17 h.mg)	Arylsulfatase A (nmol/17 h.mg)
Patient‐1	219.42	662.71	92.24	1.05	37.55
Patient‐2	283.26	852.63	56.58	3.42	61.32
Control‐1	207.09	696.68	80.08	2.80	53.70
Control‐2	152.65	723.32	99.63	1.74	41.25
Control‐3	160.05	652.30	69.05	3.05	34.55
Control‐4	198.90	728.19	88.24	2.70	40.31
Control‐5	227.42	782.59	99.94	1.84	46.61
Control‐6	264.70	880.85	132.69	3.15	62.29

### 3.5 Accumulation of lamellated membrane structures, deformed mitochondria, and lipid droplets in patients' EBV‐LCLs

**FIGURE 6:**
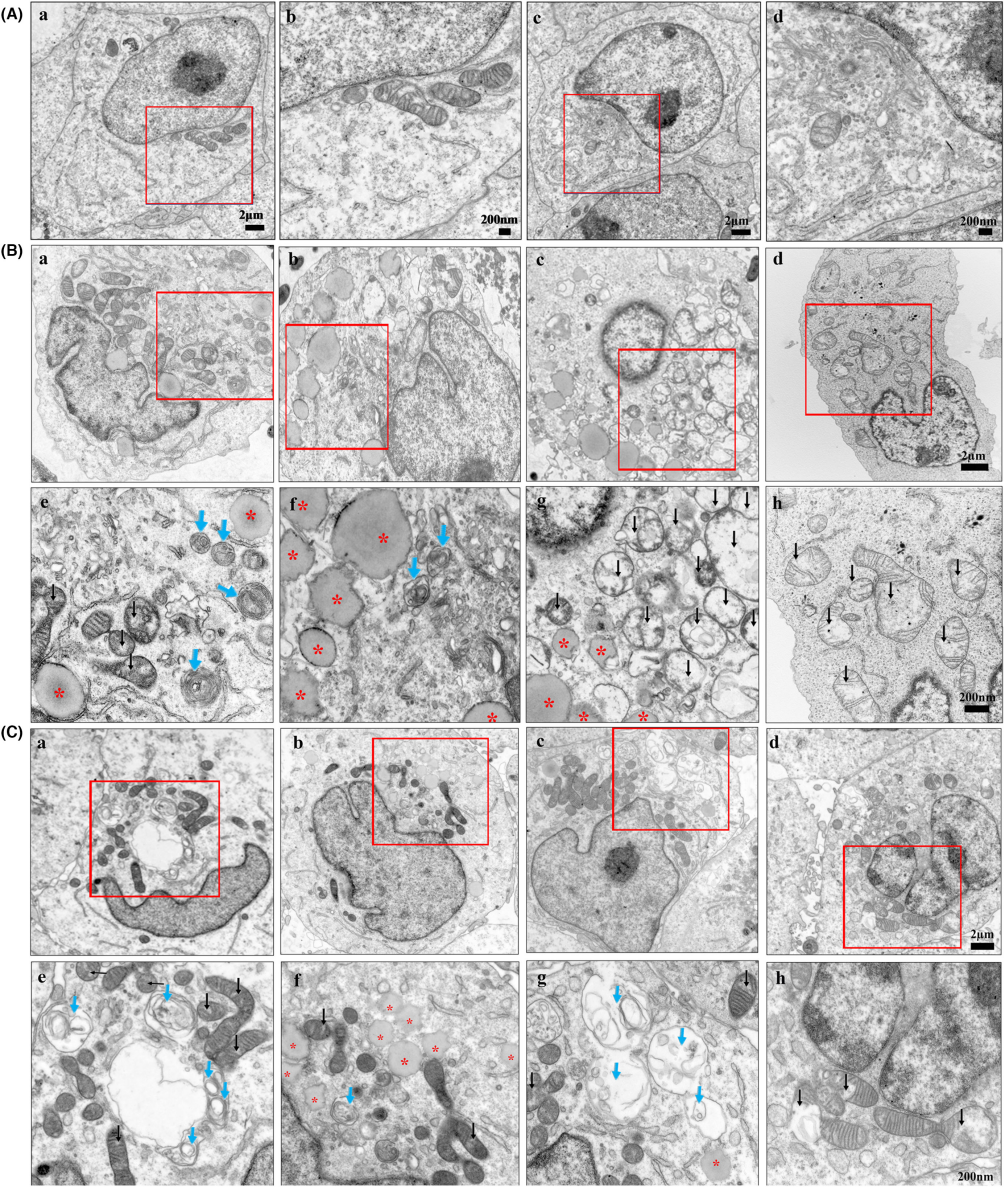
Electron micrographs. (A) Ultrastructure of EBV‐LCLs from health controls. Boxes in (a, c) indicate area shown at higher magnification in (b, d). Scale bars: a, c = 2 μm; b, d = 200 nm. (B, C) Ultrastructure of EBV‐LCLs from patient 1 (B) and patient 2 (C). Boxes in (a–d) indicate area shown at higher magnification in (e–h). Scale bars: a–d = 2 μm; e–h = 200 nm. Electron micrographs showed an accumulation of lamellated membrane structures (blue arrows), deformed mitochondria (black arrows), and lipid droplets (red asterisks) in patient EBV‐LCLs.

Electron microscopy studies of *Slc9a6* knockout brain exhibited an abnormal distribution of vesicular organelles and lamellar bodies.^11^ In order to test for the possibility of using electron microscopy analysis for the diagnosis of CS, we performed electron microscopy analysis of EBV‐LCLs from our two patients and two controls. Similar to the results from *Slc9a6* ^*−/−*^ mice,^11^ electron micrographs of the EBV‐LCLs from our two patients revealed an accumulation of lamellated membrane structures resembling storage bodies observed in some lysosomal diseases (Figure 6). Furthermore, various alterations of mitochondrial morphology were seen in patient EBV‐LCLs, such as swollen mitochondria with disrupted cristae, small vacuolations, and ruptures of the outer membrane (Figure 6). Moreover, both the number and size of lipid droplets were markedly increased in the cytoplasm of patient EBV‐LCLs, suggesting an accumulation of lipid droplets in patient EBV‐LCLs (Figure 6).

## 4 DISCUSSION

Deleterious variants in the *SLC9A6* gene underly the rare X‐linked neurodevelopmental and neurodegenerative disorder CS.^1^, ^2^, ^3^, ^4^, ^5^ The protein encoded by *SLC9A6*, NHE6, predominantly localizes to early and recycling endosomes where it plays a role in regulating the luminal pH as well as in endosomal trafficking and signaling.^2^, ^13^, ^23^, ^24^ To date, more than 80 different mutations in *SLC9A6* have been identified in CS patients, including frameshift, nonsense, missense, splicing, and deletion variants.^5^, ^8^, ^25^, ^26^ In this study, we describe two unrelated patients who showed many of the core symptoms of CS, such as severe developmental delay, absence of speech, ataxia, hyperkinetic behavior, intractable epilepsy, and microcephaly. Both probands harbored previously reported frameshift variants in *SLC9A6* (p.T521Yfs*23, p.H203Lfs*10).^22^ It is unclear whether the two variants are causative, because they have not been evaluated for functional effects. To confirm the pathogenicity of p.T521Yfs*23 and p.H203Lfs*10 variants, we firstly performed qRT‐PCR and western blot analysis on lymphoblastoid cells from the two patients. This revealed that the two *SLC9A6* frameshift variants resulted in significantly decreased mRNA levels and lack of normal NHE6 protein. These findings conclusively indicate that p.T521Yfs*23 and p.H203Lfs*10 variants should cause total loss of function and further confirm that CS in humans most likely arise from NHE6 loss of function.

Patients with CS share common clinical manifestations, such as developmental delay, lack of speech, ataxia, epilepsy, and postnatal microcephaly. However, these clinical presentations are not specific for CS and therefore, CS patients often present a diagnostic challenge. At the present time, the diagnosis of CS is based on suggestive clinical features and pathogenic variants in *SLC9A6* identified by molecular genetic testing, but sometimes diagnosis based on mutation analysis can be inconclusive in patients suspected of CS because a number of genetic variants are of unknown significance according to the American College of Medical Genetics and Genomics guideline. The accurate prediction of the effect of novel *SLC9A6* variants on CS risk is an unmet need in clinical practice. Until now, there is no objective diagnostic tool to demonstrate the significance of single *SLC9A6* variants. Therefore, in addition to gathering further insight into the disease mechanism, we aimed at identifying complementary methods in the diagnosis of CS. To this end, we analyzed patient and control EBV‐LCLs for cholesterol accumulation, lysosomal enzyme activities and ultrastructural morphology.

Previous studies demonstrated lysosomal dysfunction as neuropathological hallmarks of NHE6‐deficient mouse brains, as evident from the accumulation of GM2 ganglioside.^11^, ^27^ Consistently, GM2‐accumulating neurons of *Slc9a6* ^−/−^ mice exhibited little or no detectable β‐hexosaminidase activity.^11^ Therefore, we tested the activities of five lysosomal enzyme activities (β‐hexosaminidase A, β‐hexosaminidase A + B, β‐galactosidase, galactocerebrosidase, and arylsulfatase A) in EBV‐LCLs from our two patients. However, we found no significant alteration in lysosomal enzyme activities, showing that lysosomal enzyme activities in lymphoblastoid cells should not be used for the diagnosis of CS.

As staining of *Slc9a6* ^*−/−*^ mouse brain with the fluorescent probe filipin demonstrated abnormal accumulation of unesterified cholesterol in late endosomes and lysosomes of certain neurons,^11^ we stained lymphoblastoid cells of the patients and controls with filipin. We found a statistically significant increase in fluorescence intensity in patient 1, but only a slight, non‐significant increase in patient 2 when compared to controls. Therefore, we propose the use of the “filipin test” on EBV‐LCLs as a screening test in patients with a strong suspicion of CS, however, its sensitivity of is not optimal. Further evaluation with larger cohorts is required to determine the sensitivity of filipin staining in patients with CS.

Neurons of the amygdala in *Slc9a6* ^*−/−*^ mice contained various lamellar bodies.^11^ Consistent with these results, our electron microscopic ultrastructural examination of EBV‐LCLs from both patients revealed the existence of multi‐lamellar membrane structures resembling storage bodies observed in some lysosomal diseases. Lamellar bodies are specialized lipid storage or secretory organelles which may contain lysosomal enzymes and apolipoproteins.^28^ Dysregulated lipid metabolism can become toxic and trigger increased oxidative stress, eventually leading to cell death. Therefore, it can be assumed that lipid metabolism defects promote the progression of neurodegeneration in CS. Moreover, electron micrographs of patient EBV‐LCLs demonstrated a marked increase in number and size of lipid droplets, although an increase in lipid droplets was previously not reported in the NHE6‐null mouse brain^11^ or in human postmortem studies of CS.^8^ These findings, in addition to the accumulation of unesterified cholesterol in EBV‐LCLs from patient 1, support that NHE6 loss of function leads to a disturbance in lipid metabolism beyond reduced GM2 degradation described in NHE6‐deficeint neurons.^11^

The analysis of a rat model showed that lysosome dysfunction is followed by autophagy defects in NHE6‐deficient brain.^27^ Mitochondrial morphology directly reflects the function and health of the mitochondria, whose quality control is especially dependent on efficient autophagy‐lysosome function.^29^, ^30^ To date, no clinical data demonstrated defects in mitochondrial integrity in patients with CS. Our study is the first time to show a marked alteration in mitochondrial morphology, such as swollen mitochondria with disrupted cristae, small vacuolations, and a rupture of the outer mitochondrial membrane in EBV‐LCLs from both patients with CS. Beyond energy production, mitochondrial are also involved in neurotransmitter metabolism, reactive oxygen species (ROS) production, calcium homeostasis, cell survival, and death regulation.^29^, ^30^ Neurons are particularly vulnerable to mitochondrial defects, which are implicated in many neurodegenerative diseases,^31^, ^32^, ^33^ such as Parkinson's disease,^34^, ^35^ Alzheimer's disease,^36^, ^37^ Huntington's disease,^38^ and amyotrophic lateral sclerosis.^39^, ^40^ Sustained mitochondrial damage leads to decreased ATP synthesis, increased ROS production, and reduced calcium buffering, all of which result in neuronal loss characteristic of neurodegenerative diseases.^41^ Hence, mitochondrial defects may play a role in the development and/or progression of neurodegeneration in CS.

In addition to hinting at dysregulated lipid metabolism and mitochondrial dysfunction in CS, our data suggest that electron microscopy examination of patient EBV‐LCLs has a high potential as a CS screening assay in clinical practice. Together with filipin staining, this analysis of patient lymphoblastoid cells could be a useful non‐invasive in vitro tool to improve variant interpretation in CS. In order to more accurately assess the diagnostic value of filipin staining and electron microscopy in CS, more in‐depth investigations with larger cohorts will be required. However, the rarity of this disease poses a challenge for patient enrollment and sample collection.

In summary, we present two male pediatric patients with CS caused by two reported frameshift variants in *SLC9A6*. We suggest that CS in humans most likely arise from NHE6 loss of function, and alterations of mitochondrial and lipid metabolism may play a role in the pathogenesis of CS. Moreover, this study provides the first evidence that the combination of filipin staining with electron microscopy analysis of patient lymphoblastoid cells allows specific confirmation of a clinically assumed or suspected diagnosis of CS.

## AUTHOR CONTRIBUTIONS

X.Z., H.H., and J.P. conceived and designed this project. X.Z., H.H., H.Z., and F.Y. examined patients and collected data. H.H. and X.Z. performed experiments and acquired data. X.Z., H.H., H.C., F.H., F.Y., T.S., and J.P. contributed to the interpretation of the results. X.Z. and H.H. wrote the manuscript. T.S. and J.P. revised the manuscript. All authors read and approved the final manuscript.

## FUNDING INFORMATION

The project was supported by grants from the National Natural Science Foundation of China (82,071,462, 81,771,409 to PJ and 82,201,316 to HH), the Natural Science Foundation of Hunan Province (2022JJ40785 to HH), and the Key R & D Program of Hunan Province (2022SK2036 to PJ).

## CONFLICT OF INTEREST STATEMENT

The authors declare no conflicts of interest.

## Data Availability

The data that support the findings of this study are available from the corresponding author upon reasonable request.

## References

[1] Christianson AL , Stevenson RE , van der Meyden CH , et al. X linked severe mental retardation, craniofacial dysmorphology, epilepsy, ophthalmoplegia, and cerebellar atrophy in a large south African kindred is localised to Xq24‐q27. J Med Genet. 1999;36(10):759‐766. 10528855 10.1136/jmg.36.10.759 PMC1734236

[2] Gilfillan GD , Selmer KK , Roxrud I , et al. *SLC9A6* mutations cause X‐linked mental retardation, microcephaly, epilepsy, and ataxia, a phenotype mimicking Angelman syndrome. Am J Hum Genet. 2008;82(4):1003‐1010. 18342287 10.1016/j.ajhg.2008.01.013 PMC2427207

[3] Pescosolido MF , Stein DM , Schmidt M , et al. Genetic and phenotypic diversity of NHE6 mutations in Christianson syndrome. Ann Neurol. 2014;76(4):581‐593. 25044251 10.1002/ana.24225 PMC4304796

[4] Schroer RJ , Holden KR , Tarpey PS , et al. Natural history of Christianson syndrome. Am J Med Genet A. 2010;152A(11):2775‐2783. 20949524 10.1002/ajmg.a.33093 PMC3698558

[5] Ilie A , Gao AYL , Boucher A , et al. A potential gain‐of‐function variant of *SLC9A6* leads to endosomal alkalinization and neuronal atrophy associated with Christianson syndrome. Neurobiol Dis. 2019;121:187‐204. 30296617 10.1016/j.nbd.2018.10.002

[6] Masurel‐Paulet A , Piton A , Chancenotte S , et al. A new family with an *SLC9A6* mutation expanding the phenotypic spectrum of Christianson syndrome. Am J Med Genet A. 2016;170(8):2103‐2110. 27256868 10.1002/ajmg.a.37765

[7] Sinajon P , Verbaan D , So J . The expanding phenotypic spectrum of female SLC9A6 mutation carriers: a case series and review of the literature. Hum Genet. 2016;135(8):841‐850. 27142213 10.1007/s00439-016-1675-5

[8] Garbern JY , Neumann M , Trojanowski JQ , et al. A mutation affecting the sodium/proton exchanger, *SLC9A6*, causes mental retardation with tau deposition. Brain. 2010;133(Pt 5):1391‐1402. 20395263 10.1093/brain/awq071 PMC2859154

[9] Pedersen SF , Counillon L . The *SLC9A*‐C mammalian Na(+)/H(+) exchanger family: molecules, mechanisms, and physiology. Physiol Rev. 2019;99(4):2015‐2113. 31507243 10.1152/physrev.00028.2018

[10] Xu H , Ghishan FK , Kiela PR . SLC9 gene family: function, expression, and regulation. Compr Physiol. 2018;8(2):555‐583. 29687889 10.1002/cphy.c170027 PMC6354930

[11] Strømme P , Dobrenis K , Sillitoe RV , et al. X‐linked Angelman‐like syndrome caused by *SLC9A6* knockout in mice exhibits evidence of endosomal‐lysosomal dysfunction. Brain. 2011;134(Pt 11):3369‐3383. 21964919 10.1093/brain/awr250 PMC3212719

[12] Deane EC , Ilie AE , Sizdahkhani S , das Gupta M , Orlowski J , McKinney RA . Enhanced recruitment of endosomal Na+/H+ exchanger NHE6 into dendritic spines of hippocampal pyramidal neurons during NMDA receptor‐dependent long‐term potentiation. J Neurosci. 2013;33(2):595‐610. 23303939 10.1523/JNEUROSCI.2583-12.2013 PMC6704919

[13] Ouyang Q , Lizarraga SB , Schmidt M , et al. Christianson syndrome protein NHE6 modulates TrkB endosomal signaling required for neuronal circuit development. Neuron. 2013;80(1):97‐112. 24035762 10.1016/j.neuron.2013.07.043 PMC3830955

[14] Brett CL , Wei Y , Donowitz M , Rao R . Human Na(+)/H(+) exchanger isoform 6 is found in recycling endosomes of cells, not in mitochondria. Am J Physiol Cell Physiol. 2002;282(5):C1031‐C1041. 11940519 10.1152/ajpcell.00420.2001

[15] Nakamura N , Tanaka S , Teko Y , Mitsui K , Kanazawa H . Four Na+/H+ exchanger isoforms are distributed to Golgi and post‐Golgi compartments and are involved in organelle pH regulation. J Biol Chem. 2005;280(2):1561‐1572. 15522866 10.1074/jbc.M410041200

[16] Peng J , Wang Y , He F , et al. Novel west syndrome candidate genes in a Chinese cohort. CNS Neurosci Ther. 2018;24(12):1196‐1206. 29667327 10.1111/cns.12860 PMC6489871

[17] Den Tandt WR , Scharpe S . Characteristics of hexosaminidase a in homogenates of white blood cells using methylumbelliferyl‐N‐acetyl‐beta‐D‐glucosaminide‐6‐sulphate as substrate. Clin Chim Acta. 1991;199(3):231‐236. 1837501 10.1016/0009-8981(91)90116-t

[18] Perez LF , Tutor JC . Assay of beta‐N‐acetylhexosaminidase isoenzymes in different biological specimens by means of determination of their activation energies. Clin Chem. 1998;44(2):226‐231. 9474016

[19] Anderson JK , Mole JE , Baker HJ . Purification and characterization of GM1 ganglioside beta‐galactosidase from normal feline liver and brain. Biochemistry. 1978;17(3):467‐473. 413573 10.1021/bi00596a015

[20] Wiederschain G , Raghavan S , Kolodny E . Characterization of 6‐hexadecanoylamino‐4‐methylumbelliferyl‐beta‐D‐galactopyranoside as fluorogenic substrate of galactocerebrosidase for the diagnosis of Krabbe disease. Clin Chim Acta. 1992;205(1–2):87‐96. 1521344 10.1016/s0009-8981(05)80003-8

[21] Lee‐Vaupel M , Conzelmann E . A simple chromogenic assay for arylsulfatase a. Clin Chim Acta. 1987;164(2):171‐180. 2885112 10.1016/0009-8981(87)90068-4

[22] Riess A , Rossier E , Krüger R , et al. Novel *SLC9A6* mutations in two families with Christianson syndrome. Clin Genet. 2013;83(6):596‐597. 22931061 10.1111/j.1399-0004.2012.01948.x

[23] Pescosolido MF , Ouyang Q , Liu JS , Morrow EM . Loss of Christianson syndrome Na(+)/H(+) exchanger 6 (NHE6) causes abnormal endosome maturation and trafficking underlying lysosome dysfunction in neurons. J Neurosci. 2021;41(44):9235‐9256. 34526390 10.1523/JNEUROSCI.1244-20.2021 PMC8570832

[24] Freeman SA , Grinstein S , Orlowski J . Determinants, maintenance, and function of organellar pH. Physiol Rev. 2023;103(1):515‐606. 35981302 10.1152/physrev.00009.2022

[25] Ieda D , Hori I , Nakamura Y , et al. A novel splicing mutation in *SLC9A6* in a boy with Christianson syndrome. Hum Genome Var. 2019;6:15. 30937176 10.1038/s41439-019-0046-x PMC6434044

[26] Ilie A , Boucher A , Park J , Berghuis AM , McKinney RA , Orlowski J . Assorted dysfunctions of endosomal alkali cation/proton exchanger *SLC9A6* variants linked to Christianson syndrome. J Biol Chem. 2020;295(20):7075‐7095. 32277048 10.1074/jbc.RA120.012614 PMC7242699

[27] Lee Y , Miller MR , Fernandez MA , et al. Early lysosome defects precede neurodegeneration with amyloid‐beta and tau aggregation in NHE6‐null rat brain. Brain. 2022;145(9):3187‐3202. 34928329 10.1093/brain/awab467 PMC10147331

[28] Schmitz G , Muller G . Structure and function of lamellar bodies, lipid‐protein complexes involved in storage and secretion of cellular lipids. J Lipid Res. 1991;32(10):1539‐1570. 1797938

[29] Burte F , Carelli V , Chinnery PF , Yu‐Wai‐Man P . Disturbed mitochondrial dynamics and neurodegenerative disorders. Nat Rev Neurol. 2015;11(1):11‐24. 25486875 10.1038/nrneurol.2014.228

[30] Giacomello M , Pyakurel A , Glytsou C , Scorrano L . The cell biology of mitochondrial membrane dynamics. Nat Rev Mol Cell Biol. 2020;21(4):204‐224. 32071438 10.1038/s41580-020-0210-7

[31] Gan L , Cookson MR , Petrucelli L , La Spada AR . Converging pathways in neurodegeneration, from genetics to mechanisms. Nat Neurosci. 2018;21(10):1300‐1309. 30258237 10.1038/s41593-018-0237-7 PMC6278826

[32] Gorman GS , Chinnery PF , DiMauro S , et al. Mitochondrial diseases. Nat Rev Dis Primers. 2016;2:16080. 27775730 10.1038/nrdp.2016.80

[33] Murphy MP , Hartley RC . Mitochondria as a therapeutic target for common pathologies. Nat Rev Drug Discov. 2018;17(12):865‐886. 30393373 10.1038/nrd.2018.174

[34] Bose A , Beal MF . Mitochondrial dysfunction in Parkinson's disease. J Neurochem. 2016;139(Suppl 1):216‐231. 27546335 10.1111/jnc.13731

[35] Rocha EM , De Miranda B , Sanders LH . Alpha‐synuclein: pathology, mitochondrial dysfunction and neuroinflammation in Parkinson's disease. Neurobiol Dis. 2018;109(Pt B):249‐257. 28400134 10.1016/j.nbd.2017.04.004

[36] Shoshan‐Barmatz V , Nahon‐Crystal E , Shteinfer‐Kuzmine A , Gupta R . VDAC1, mitochondrial dysfunction, and Alzheimer's disease. Pharmacol Res. 2018;131:87‐101. 29551631 10.1016/j.phrs.2018.03.010

[37] Wang W , Zhao F , Ma X , Perry G , Zhu X . Mitochondria dysfunction in the pathogenesis of Alzheimer's disease: recent advances. Mol Neurodegener. 2020;15(1):30. 32471464 10.1186/s13024-020-00376-6 PMC7257174

[38] Carmo C , Naia L , Lopes C , Rego AC . Mitochondrial dysfunction in Huntington's disease. Adv Exp Med Biol. 2018;1049:59‐83. 29427098 10.1007/978-3-319-71779-1_3

[39] Wiedemann FR , Manfredi G , Mawrin C , Beal MF , Schon EA . Mitochondrial DNA and respiratory chain function in spinal cords of ALS patients. J Neurochem. 2002;80(4):616‐625. 11841569 10.1046/j.0022-3042.2001.00731.x

[40] Smith EF , Shaw PJ , De Vos KJ . The role of mitochondria in amyotrophic lateral sclerosis. Neurosci Lett. 2019;710:132933. 28669745 10.1016/j.neulet.2017.06.052

[41] Norat P , Soldozy S , Sokolowski JD , et al. Mitochondrial dysfunction in neurological disorders: exploring mitochondrial transplantation. NPJ Regen Med. 2020;5(1):22. 33298971 10.1038/s41536-020-00107-x PMC7683736

